# Poly[[μ-chlorido-μ-[2-(2,4-di­fluoro­phen­yl)-1,3-bis­(1,2,4-triazol-1-yl)propan-2-ol-κ^2^ *N* ^4^:*N* ^4′^]-zinc] chloride dihydrate]

**DOI:** 10.1107/S1600536813026524

**Published:** 2013-10-02

**Authors:** Gang-Hong Pan, Jin-Niu Tang, Shi-Hua Xu, Zhong-Jing Huang, Bo-Fa Mo

**Affiliations:** a College of Chemistry and Chemical Engineering, Guangxi University for Nationalities, Nanning 530006, People’s Republic of China

## Abstract

The title compound, {[ZnCl(C_13_H_12_F_2_N_6_O)_2_]Cl·2H_2_O}_*n*_, is a two-dimensional coordination polymer. The Zn^II^ atom is six-coordinated by four N atoms from four 2-(2,4-di­fluoro­phen­yl)-1,3-bis­(1,2,4-triazol-1-yl)propan-2-ol (HFlu) ligands and by two Cl atoms in a distorted octa­hedral geometry. Two Cl atoms bridge two Zn^II^ atoms, forming a centrosymmetric dinuclear unit. The HFlu ligands connect the dinuclear units into a 4^4^ net parallel to (001) when the dinuclear unit is considered as a node. O—H⋯O and O—H⋯Cl hydrogen bonds link the cationic layer, free chloride anions and lattice water mol­ecules. Intra­layer π–π inter­actions between the triazole rings are observed [centroid–centroid distance = 3.716 (6) Å].

## Related literature

For background to this class of compounds, see: Han *et al.* (2006*a* ▶,*b* ▶). For related structures, see: Gao *et al.* (2001 ▶); Zhang *et al.* (2007 ▶).
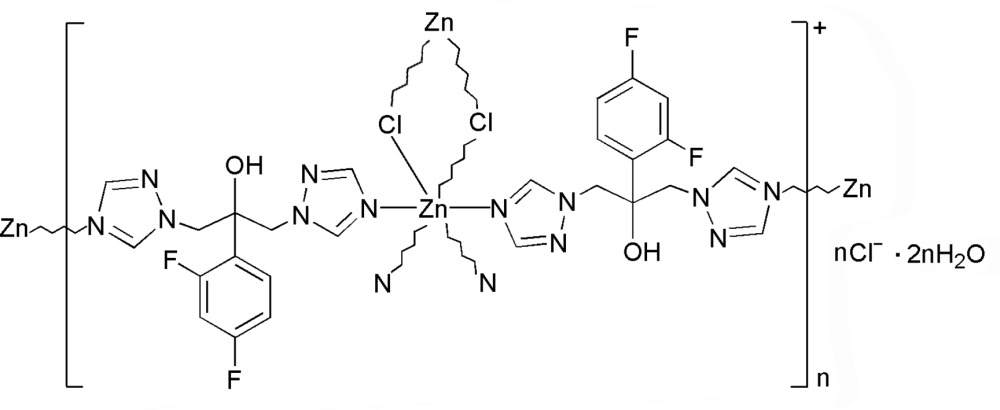


## Experimental

### 

#### Crystal data

[ZnCl(C_13_H_12_F_2_N_6_O)_2_]Cl·2H_2_O
*M* *_r_* = 784.89
Triclinic, 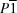

*a* = 10.2310 (6) Å
*b* = 11.8118 (6) Å
*c* = 14.3588 (9) Å
α = 91.191 (7)°
β = 107.481 (5)°
γ = 106.074 (6)°
*V* = 1580.11 (18) Å^3^
*Z* = 2
Mo *K*α radiation
μ = 1.03 mm^−1^
*T* = 296 K
0.25 × 0.25 × 0.21 mm

#### Data collection

Bruker SMART 1000 CCD diffractometer
Absorption correction: multi-scan (*SADABS*; Bruker, 2001 ▶) *T*_min_ = 0.784, *T*_max_ = 0.813
8374 measured reflections
5465 independent reflections
3137 reflections with *I* > 2σ(*I*)
*R*_int_ = 0.064

#### Refinement

*R*[*F* ^2^ > 2σ(*F* ^2^)] = 0.080
*wR*(*F* ^2^) = 0.306
*S* = 1.07
5465 reflections
444 parameters
H-atom parameters constrained
Δρ_max_ = 0.92 e Å^−3^
Δρ_min_ = −1.09 e Å^−3^

### 

Data collection: *SMART* (Bruker, 2007 ▶); cell refinement: *SAINT* (Bruker, 2007 ▶); data reduction: *SAINT*; program(s) used to solve structure: *SHELXS97* (Sheldrick, 2008 ▶); program(s) used to refine structure: *SHELXL97* (Sheldrick, 2008 ▶); molecular graphics: *DIAMOND* (Brandenburg, 1999 ▶); software used to prepare material for publication: *SHELXTL* (Sheldrick, 2008 ▶).

## Supplementary Material

Crystal structure: contains datablock(s) I. DOI: 10.1107/S1600536813026524/hy2637sup1.cif

Structure factors: contains datablock(s) I. DOI: 10.1107/S1600536813026524/hy2637Isup2.hkl

Additional supplementary materials:  crystallographic information; 3D view; checkCIF report

## Figures and Tables

**Table 1:** Hydrogen-bond geometry (Å, °)

*D*—H⋯*A*	*D*—H	H⋯*A*	*D*⋯*A*	*D*—H⋯*A*
O1—H1⋯Cl2^i^	0.82	2.29	3.103 (7)	172
O2—H2⋯O4^i^	0.82	1.87	2.653 (9)	160
O3—H3*A*⋯Cl2^ii^	0.85	2.32	3.163 (11)	170
O3—H3*B*⋯Cl1^iii^	0.85	2.38	3.221 (10)	170
O4—H4*A*⋯O2^iv^	0.85	2.24	2.784 (9)	122
O4—H4*B*⋯Cl2	0.85	2.29	3.101 (8)	160

Symmetry codes: (i) 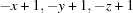; (ii) 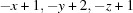; (iii) 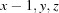; (iv) 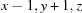.

**Fig. 1.**
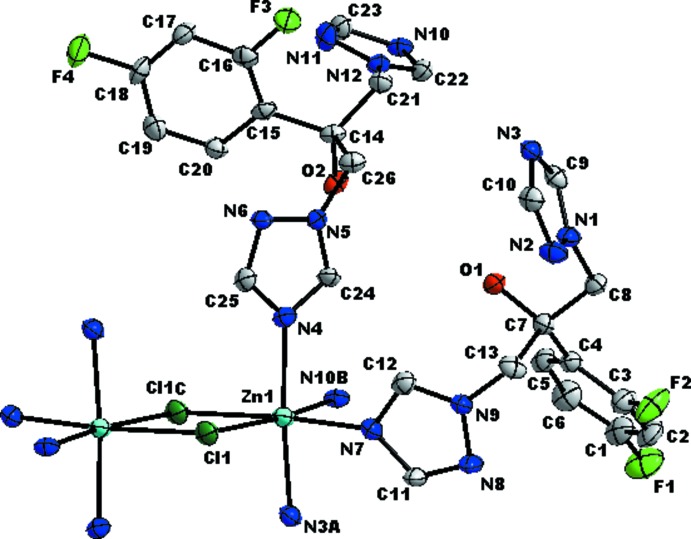
The coordination environment around the ZnII atom in the title compound (uncoordinated Cl atom, water molecules and H atoms have been omitted for clarity). Displacement ellipsoids are drawn at the 30% probability level. [Symmetry codes: (A) 1+x, y, z; (B) 2-x, -y, 1-z; (C) 3-x, 1-y, 1-z.]

**Fig. 2.**
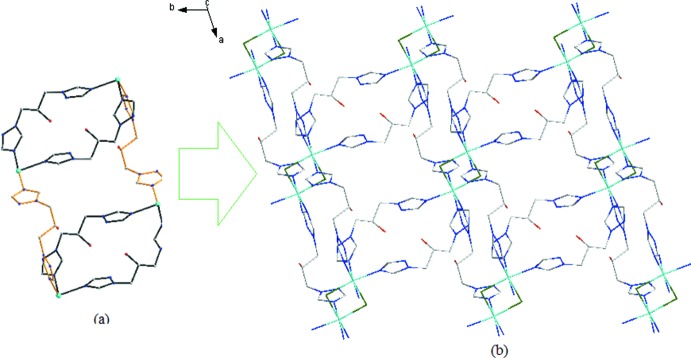
(a) A view of the grid unit in the title compound. (b) A view of the two-dimensional framework. H atoms and difluorophenyl groups of HFlu are omitted for clarity.

**Fig. 3.**
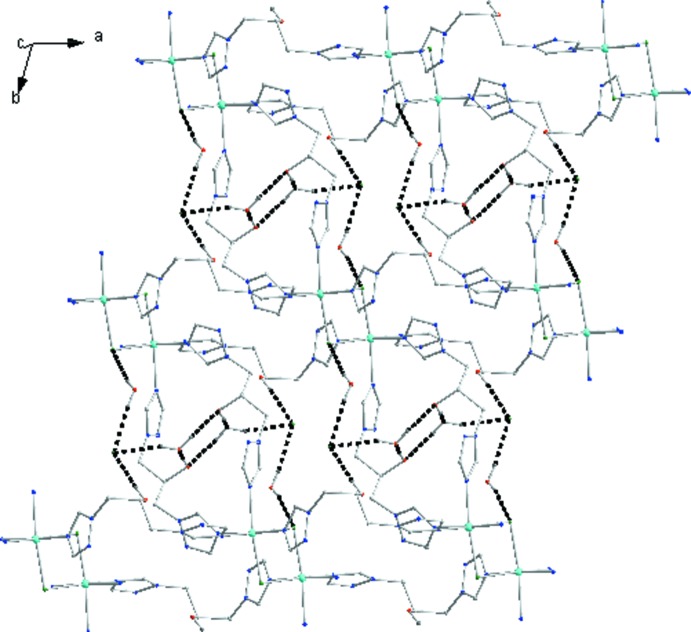
A view of hydrogen bonding interactions (dashed lines) in the title compound. H atoms and difluorophenyl groups of HFlu are omitted for clarity.

## supplementary crystallographic information

### 1. Comment

Fluconazole 2-(2,4-difluorophenyl)-1,3-bis(1,2,4-triazol-1-yl) propan-2-ol, which is a 1,2,4-triazole derivative, is not only a widely used antifungal medicine but also a good flexible ligand to construct metal-organic polymers with optical properties and medical applications (Han *et al.*, 2006*a*,*b*). Fluconazole can coordinate to metal ions in different configurations. We here report a new coordination polymer based on fluconazole.

The asymmetric unit of the title compound contains one Zn^II^ ion, two 2-(2,4-difluorophenyl)-1,3-bis(1,2,4-triazol-1-yl)propan-2-ol (HFlu) ligands, one coordinated Cl^-1^ anion, one free Cl^-1^ anion and two free water molecules. As shown in Fig. 1, the Zn^II^ ion is six-coordinated by four N atoms from four HFlu ligands and two bridging Cl^-1^ anions. The Zn—N bond lengths range from 2.127 (8) to 2.197 (7) Å, and the Zn—Cl bond distances are 2.418 (3) and 2.732 (3) Å. The Zn—N bond lengths are in the normal range as observed in other Zn(II) complexes (Zhang *et al.*, 2007). However, the Zn—Cl bond distances are longer than those as observed in other Zn(II) complexe (Gao *et al.*, 2001). Two Zn^II^ ions are connected by two HFlu ligands, forming a Zn_2_(HFlu)_2_ macrocycle, in which the Zn···Zn distance is 11.297 (2)Å. The other two HFlu ligands link the macrocycle along the *a* axis with a Zn···Zn distance of 10.231 (2) Å to form a grid unit with dimensions of 11.30 × 10.23 Å^2^ (Fig. 2a). These grid units are further connected by two Cl^-1^ anions with a Zn···Zn distance of 3.879 (1) Å into a two-dimensional structure (Fig. 2b), in which another type of grid with dimensions of 10.231 (2) × 3.879 (1) Å^2^ is formed.

Free water molecules and free Cl^-1^ anions are accommodated in the residual empties to shrink the void space and stabilize the structure. Moreover, there are intermolecular O—H···O and O—H···Cl hydrogen bonds involving the cationic layer, free Cl^-1^ anions and water molecules (Table 1, Fig. 3). These interactions further stabilize the structural framework.

### 2. Experimental

A mixture of fluconazole (153 mg, 0.5 mmol), ZnCl_2_ (136 mg, 1.0 mmol), 15 ml H_2_O, and 3 ml ethanol was placed in a Parr Teflon-lined stainless steel vessel (30 ml), and then the vessel was sealed and heated at 423 K for 3 days. After the mixture was slowly cooled to room temperature, colorless block-shaped crystals of the title compound was obtained. Analysis, calculated for C_26_H_28_Cl_2_F_4_N_12_O_4_Zn: C 39.79, H 3.60, N 21.42%; found: C 39.66, H 3.52, N 21.28%.

### 3. Refinement

H atoms were positioned geometrically and refined as riding atoms, with C—H = 0.93 (aromatic), 0.97(methylene) and O—H = 0.82 Å and with *U*_iso_(H) = 1.2(1.5 for hydroxyl)*U*_eq_(C,O). H atoms of the water molecules were located in a difference Fourier map and refined as riding, with O—H = 0.85 Å and *U*_iso_(H) = 1.2*U*_eq_(O). The maximum remaining electron density was found 1.00 Å from Zn1 and the minimum density 0.83 Å from Zn1.

### Crystal data

**Table:**

[ZnCl(C_13_H_12_F_2_N_6_O)_2_]Cl·2H_2_O	*Z* = 2
*M**_r_* = 784.89	*F*(000) = 800
Triclinic, *P*1	*D*_x_ = 1.650 Mg m^−^^3^
Hall symbol: -P 1	Mo *K*α radiation, λ = 0.71073 Å
*a* = 10.2310 (6) Å	Cell parameters from 859 reflections
*b* = 11.8118 (6) Å	θ = 2.2–22.1°
*c* = 14.3588 (9) Å	µ = 1.03 mm^−^^1^
α = 91.191 (7)°	*T* = 296 K
β = 107.481 (5)°	Block, colorless
γ = 106.074 (6)°	0.25 × 0.25 × 0.21 mm
*V* = 1580.11 (18) Å^3^	

### Data collection

**Table:**

Bruker SMART 1000 CCD diffractometer	5465 independent reflections
Radiation source: fine-focus sealed tube	3137 reflections with *I* > 2σ(*I*)
Graphite monochromator	*R*_int_ = 0.064
φ and ω scans	θ_max_ = 25.0°, θ_min_ = 1.5°
Absorption correction: multi-scan (*SADABS*; Bruker, 2001)	*h* = −12→12
*T*_min_ = 0.784, *T*_max_ = 0.813	*k* = −14→12
8374 measured reflections	*l* = −14→17

### Refinement

**Table:**

Refinement on *F*^2^	Primary atom site location: structure-invariant direct methods
Least-squares matrix: full	Secondary atom site location: difference Fourier map
*R*[*F*^2^ > 2σ(*F*^2^)] = 0.080	Hydrogen site location: inferred from neighbouring sites
*wR*(*F*^2^) = 0.306	H-atom parameters constrained
*S* = 1.07	*w* = 1/[σ^2^(*F*_o_^2^) + (0.1513*P*)^2^ + 4.0805*P*] where *P* = (*F*_o_^2^ + 2*F*_c_^2^)/3
5465 reflections	(Δ/σ)_max_ < 0.001
444 parameters	Δρ_max_ = 0.92 e Å^−^^3^
0 restraints	Δρ_min_ = −1.09 e Å^−^^3^

### Special details

**Table:**

Geometry. All esds (except the esd in the dihedral angle between two l.s. planes) are estimated using the full covariance matrix. The cell esds are taken into account individually in the estimation of esds in distances, angles and torsion angles; correlations between esds in cell parameters are only used when they are defined by crystal symmetry. An approximate (isotropic) treatment of cell esds is used for estimating esds involving l.s. planes.
Refinement. Refinement of F^2^ against ALL reflections. The weighted R-factor wR and goodness of fit S are based on F^2^, conventional R-factors R are based on F, with F set to zero for negative F^2^. The threshold expression of F^2^ > 2sigma(F^2^) is used only for calculating R-factors(gt) etc. and is not relevant to the choice of reflections for refinement. R-factors based on F^2^ are statistically about twice as large as those based on F, and R- factors based on ALL data will be even larger.

### Fractional atomic coordinates and isotropic or equivalent isotropic displacement parameters (Å^2^)

**Table:**

	*x*	*y*	*z*	*U*_iso_*/*U*_eq_	
Zn1	1.32995 (12)	0.41377 (10)	0.39336 (8)	0.0404 (4)	
Cl1	1.4508 (3)	0.6199 (2)	0.45651 (17)	0.0448 (6)	
Cl2	0.4395 (3)	0.9322 (2)	0.6822 (2)	0.0584 (8)	
F1	0.7971 (10)	−0.0762 (7)	−0.0678 (5)	0.086 (2)	
F2	0.6122 (9)	0.2436 (7)	−0.0664 (4)	0.083 (2)	
F3	0.8098 (7)	0.2576 (6)	0.7540 (4)	0.0657 (18)	
F4	1.2544 (8)	0.3386 (7)	0.9953 (4)	0.079 (2)	
N1	0.5148 (8)	0.3554 (8)	0.1793 (5)	0.043 (2)	
N2	0.5348 (9)	0.4724 (7)	0.1718 (6)	0.048 (2)	
N3	0.4536 (8)	0.4152 (7)	0.2976 (5)	0.041 (2)	
N4	1.1903 (9)	0.4025 (7)	0.4804 (5)	0.041 (2)	
N5	1.0061 (8)	0.3403 (6)	0.5307 (5)	0.0335 (18)	
N6	1.0891 (8)	0.4430 (6)	0.5911 (5)	0.0345 (18)	
N7	1.1616 (8)	0.4340 (7)	0.2632 (5)	0.041 (2)	
N8	1.0182 (9)	0.4016 (9)	0.1051 (6)	0.054 (2)	
N9	0.9478 (8)	0.4220 (7)	0.1671 (5)	0.0388 (19)	
N10	0.7509 (8)	−0.2201 (7)	0.6379 (5)	0.0393 (19)	
N11	0.8367 (9)	−0.0419 (7)	0.7239 (6)	0.048 (2)	
N12	0.7677 (8)	−0.0339 (7)	0.6293 (5)	0.0381 (19)	
O1	0.7534 (7)	0.2765 (6)	0.2468 (4)	0.0446 (17)	
H1	0.6956	0.2212	0.2602	0.067*	
O2	0.9802 (7)	0.1089 (6)	0.5651 (4)	0.0417 (16)	
H2	0.9323	0.0797	0.5087	0.063*	
O3	0.5759 (12)	0.8041 (10)	0.3197 (8)	0.106 (4)	
H3A	0.5660	0.8728	0.3247	0.127*	
H3B	0.5394	0.7628	0.3585	0.127*	
O4	0.1267 (8)	0.9438 (6)	0.6285 (5)	0.055 (2)	
H4A	0.0960	0.9921	0.6543	0.065*	
H4B	0.2165	0.9582	0.6394	0.065*	
C1	0.7769 (15)	0.0150 (10)	−0.0176 (9)	0.061 (3)	
C2	0.7008 (14)	0.0812 (11)	−0.0685 (8)	0.061 (3)	
H2A	0.6611	0.0669	−0.1366	0.073*	
C3	0.6831 (12)	0.1716 (11)	−0.0163 (8)	0.055 (3)	
C4	0.7305 (10)	0.1914 (8)	0.0866 (6)	0.037 (2)	
C5	0.8047 (12)	0.1152 (9)	0.1326 (7)	0.048 (3)	
H5	0.8379	0.1228	0.2009	0.058*	
C6	0.8309 (14)	0.0295 (9)	0.0817 (8)	0.059 (3)	
H6	0.8848	−0.0178	0.1146	0.071*	
C7	0.7064 (10)	0.2849 (8)	0.1446 (6)	0.038 (2)	
C8	0.5481 (11)	0.2778 (9)	0.1112 (7)	0.044 (2)	
H8A	0.5219	0.3018	0.0456	0.053*	
H8B	0.4911	0.1963	0.1083	0.053*	
C9	0.4661 (10)	0.3227 (9)	0.2527 (7)	0.043 (2)	
H9	0.4441	0.2460	0.2701	0.051*	
C10	0.4963 (11)	0.5041 (9)	0.2440 (7)	0.045 (2)	
H10	0.4978	0.5816	0.2584	0.054*	
C11	1.1463 (11)	0.4096 (10)	0.1657 (7)	0.050 (3)	
H11	1.2202	0.3997	0.1446	0.060*	
C12	1.0311 (11)	0.4394 (8)	0.2601 (7)	0.042 (2)	
H12	1.0035	0.4532	0.3144	0.051*	
C13	0.7955 (10)	0.4106 (9)	0.1330 (7)	0.045 (3)	
H13A	0.7669	0.4244	0.0644	0.054*	
H13B	0.7766	0.4699	0.1706	0.054*	
C14	0.9018 (10)	0.1666 (8)	0.6051 (6)	0.035 (2)	
C15	0.9956 (10)	0.2149 (8)	0.7112 (6)	0.036 (2)	
C16	0.9466 (11)	0.2552 (8)	0.7784 (7)	0.043 (2)	
C17	1.0282 (12)	0.2959 (9)	0.8753 (6)	0.047 (3)	
H17	0.9900	0.3211	0.9202	0.056*	
C18	1.1686 (12)	0.2967 (10)	0.9008 (6)	0.050 (3)	
C19	1.2280 (12)	0.2644 (9)	0.8356 (7)	0.052 (3)	
H19	1.3248	0.2691	0.8550	0.062*	
C20	1.1427 (10)	0.2248 (8)	0.7411 (7)	0.040 (2)	
H20	1.1826	0.2039	0.6955	0.048*	
C21	0.7561 (10)	0.0805 (8)	0.5970 (7)	0.043 (2)	
H21A	0.7120	0.1149	0.6367	0.052*	
H21B	0.6944	0.0683	0.5292	0.052*	
C22	0.7170 (11)	−0.1387 (9)	0.5789 (7)	0.045 (3)	
H22	0.6653	−0.1546	0.5122	0.054*	
C23	0.8252 (11)	−0.1510 (9)	0.7257 (7)	0.046 (3)	
H23	0.8644	−0.1822	0.7827	0.056*	
C24	1.0684 (10)	0.3188 (8)	0.4666 (6)	0.038 (2)	
H24	1.0314	0.2539	0.4187	0.046*	
C25	1.1980 (11)	0.4760 (8)	0.5568 (6)	0.038 (2)	
H25	1.2742	0.5445	0.5829	0.045*	
C26	0.8744 (10)	0.2674 (8)	0.5427 (7)	0.038 (2)	
H26A	0.8054	0.2338	0.4787	0.046*	
H26B	0.8341	0.3160	0.5745	0.046*	

### Atomic displacement parameters (Å^2^)

**Table:**

	*U*^11^	*U*^22^	*U*^33^	*U*^12^	*U*^13^	*U*^23^
Zn1	0.0408 (7)	0.0385 (7)	0.0419 (7)	0.0027 (5)	0.0216 (5)	−0.0007 (5)
Cl1	0.0473 (14)	0.0335 (13)	0.0487 (13)	0.0027 (11)	0.0168 (11)	0.0009 (10)
Cl2	0.0528 (16)	0.0554 (17)	0.0658 (17)	0.0127 (14)	0.0199 (13)	0.0137 (13)
F1	0.126 (7)	0.067 (5)	0.085 (5)	0.039 (5)	0.054 (5)	−0.006 (4)
F2	0.127 (7)	0.099 (6)	0.043 (4)	0.071 (5)	0.020 (4)	0.013 (3)
F3	0.048 (4)	0.095 (5)	0.059 (4)	0.027 (4)	0.019 (3)	−0.005 (3)
F4	0.073 (5)	0.105 (6)	0.040 (3)	0.023 (4)	−0.002 (3)	−0.014 (3)
N1	0.037 (4)	0.057 (6)	0.040 (4)	0.018 (4)	0.017 (3)	0.006 (4)
N2	0.052 (5)	0.038 (5)	0.060 (5)	0.008 (4)	0.030 (4)	0.008 (4)
N3	0.031 (4)	0.048 (5)	0.045 (4)	0.010 (4)	0.014 (3)	0.009 (4)
N4	0.044 (5)	0.045 (5)	0.037 (4)	0.012 (4)	0.019 (3)	0.009 (4)
N5	0.035 (4)	0.034 (4)	0.031 (4)	0.007 (3)	0.012 (3)	0.002 (3)
N6	0.035 (4)	0.037 (4)	0.036 (4)	0.009 (4)	0.019 (3)	0.003 (3)
N7	0.033 (4)	0.053 (5)	0.039 (4)	0.012 (4)	0.013 (3)	0.005 (4)
N8	0.039 (5)	0.091 (7)	0.038 (4)	0.022 (5)	0.021 (4)	0.004 (4)
N9	0.031 (4)	0.051 (5)	0.037 (4)	0.010 (4)	0.015 (3)	0.007 (4)
N10	0.042 (5)	0.039 (5)	0.042 (4)	0.009 (4)	0.023 (4)	0.011 (4)
N11	0.058 (5)	0.025 (4)	0.045 (4)	−0.005 (4)	0.008 (4)	−0.001 (3)
N12	0.043 (5)	0.036 (5)	0.033 (4)	0.005 (4)	0.014 (3)	0.009 (3)
O1	0.048 (4)	0.046 (4)	0.036 (3)	0.002 (3)	0.018 (3)	−0.002 (3)
O2	0.054 (4)	0.043 (4)	0.034 (3)	0.023 (3)	0.015 (3)	0.005 (3)
O3	0.112 (9)	0.096 (8)	0.138 (9)	0.039 (7)	0.071 (7)	0.047 (7)
O4	0.060 (5)	0.052 (5)	0.051 (4)	0.025 (4)	0.010 (3)	−0.002 (3)
C1	0.083 (9)	0.036 (6)	0.061 (7)	0.006 (6)	0.031 (6)	−0.010 (5)
C2	0.080 (9)	0.064 (8)	0.036 (6)	0.018 (7)	0.020 (5)	−0.011 (5)
C3	0.057 (7)	0.067 (8)	0.045 (6)	0.023 (6)	0.017 (5)	0.011 (5)
C4	0.038 (5)	0.040 (5)	0.035 (5)	0.011 (4)	0.015 (4)	0.007 (4)
C5	0.061 (7)	0.040 (6)	0.040 (5)	0.007 (5)	0.020 (5)	−0.005 (4)
C6	0.076 (8)	0.035 (6)	0.067 (7)	0.019 (6)	0.024 (6)	0.002 (5)
C7	0.035 (5)	0.039 (5)	0.038 (5)	0.009 (4)	0.013 (4)	0.002 (4)
C8	0.046 (6)	0.047 (6)	0.039 (5)	0.007 (5)	0.020 (4)	−0.009 (4)
C9	0.035 (5)	0.043 (6)	0.053 (6)	0.014 (5)	0.014 (4)	0.005 (5)
C10	0.050 (6)	0.031 (5)	0.053 (6)	0.007 (5)	0.020 (5)	−0.002 (4)
C11	0.037 (6)	0.083 (8)	0.036 (5)	0.020 (6)	0.020 (4)	0.010 (5)
C12	0.051 (6)	0.036 (5)	0.040 (5)	0.009 (5)	0.017 (4)	0.001 (4)
C13	0.041 (6)	0.049 (6)	0.056 (6)	0.023 (5)	0.020 (5)	0.015 (5)
C14	0.042 (5)	0.036 (5)	0.037 (5)	0.015 (4)	0.023 (4)	0.008 (4)
C15	0.048 (6)	0.031 (5)	0.034 (5)	0.010 (4)	0.021 (4)	0.011 (4)
C16	0.047 (6)	0.032 (5)	0.050 (6)	0.006 (5)	0.022 (5)	0.008 (4)
C17	0.057 (7)	0.057 (7)	0.030 (5)	0.019 (5)	0.018 (4)	0.001 (4)
C18	0.063 (7)	0.058 (7)	0.023 (5)	0.019 (6)	0.005 (5)	0.006 (4)
C19	0.044 (6)	0.049 (6)	0.051 (6)	0.003 (5)	0.009 (5)	−0.001 (5)
C20	0.035 (5)	0.044 (6)	0.044 (5)	0.011 (5)	0.018 (4)	0.004 (4)
C21	0.039 (5)	0.047 (6)	0.040 (5)	0.006 (5)	0.014 (4)	0.010 (4)
C22	0.048 (6)	0.046 (6)	0.034 (5)	0.001 (5)	0.016 (4)	−0.004 (4)
C23	0.053 (6)	0.038 (6)	0.040 (5)	0.001 (5)	0.014 (5)	0.001 (4)
C24	0.041 (5)	0.037 (5)	0.037 (5)	0.002 (4)	0.021 (4)	0.006 (4)
C25	0.046 (6)	0.025 (5)	0.039 (5)	0.005 (4)	0.015 (4)	−0.002 (4)
C26	0.045 (6)	0.035 (5)	0.042 (5)	0.016 (5)	0.020 (4)	0.010 (4)

### Geometric parameters (Å, º)

**Table:**

Zn1—N3^i^	2.127 (8)	O4—H4B	0.8500
Zn1—N4	2.144 (8)	C1—C2	1.331 (16)
Zn1—N10^ii^	2.192 (8)	C1—C6	1.353 (15)
Zn1—N7	2.197 (7)	C2—C3	1.374 (15)
Zn1—Cl1	2.418 (3)	C2—H2A	0.9300
Zn1—Cl1^iii^	2.732 (3)	C3—C4	1.402 (13)
F1—C1	1.380 (13)	C4—C5	1.387 (13)
F2—C3	1.349 (12)	C4—C7	1.487 (13)
F3—C16	1.345 (12)	C5—C6	1.367 (15)
F4—C18	1.368 (11)	C5—H5	0.9300
N1—C9	1.317 (12)	C6—H6	0.9300
N1—N2	1.351 (11)	C7—C8	1.521 (13)
N1—C8	1.500 (12)	C7—C13	1.554 (13)
N2—C10	1.297 (13)	C8—H8A	0.9700
N3—C9	1.312 (13)	C8—H8B	0.9700
N3—C10	1.366 (12)	C9—H9	0.9300
N4—C24	1.317 (12)	C10—H10	0.9300
N4—C25	1.354 (12)	C11—H11	0.9300
N5—C24	1.323 (12)	C12—H12	0.9300
N5—N6	1.375 (10)	C13—H13A	0.9700
N5—C26	1.446 (12)	C13—H13B	0.9700
N6—C25	1.317 (12)	C14—C21	1.524 (13)
N7—C12	1.342 (13)	C14—C15	1.533 (12)
N7—C11	1.376 (12)	C14—C26	1.542 (11)
N8—C11	1.314 (12)	C15—C16	1.346 (14)
N8—N9	1.356 (11)	C15—C20	1.406 (13)
N9—C12	1.327 (11)	C16—C17	1.384 (13)
N9—C13	1.452 (12)	C17—C18	1.369 (15)
N10—C22	1.343 (12)	C17—H17	0.9300
N10—C23	1.367 (12)	C18—C19	1.359 (16)
N11—C23	1.262 (12)	C19—C20	1.363 (13)
N11—N12	1.350 (10)	C19—H19	0.9300
N12—C22	1.306 (12)	C20—H20	0.9300
N12—C21	1.462 (11)	C21—H21A	0.9700
O1—C7	1.415 (10)	C21—H21B	0.9700
O1—H1	0.8200	C22—H22	0.9300
O2—C14	1.415 (11)	C23—H23	0.9300
O2—H2	0.8200	C24—H24	0.9300
O3—H3A	0.8499	C25—H25	0.9300
O3—H3B	0.8500	C26—H26A	0.9700
O4—H4A	0.8500	C26—H26B	0.9700
			
N3^i^—Zn1—N4	175.2 (3)	N1—C8—C7	111.8 (8)
N3^i^—Zn1—N10^ii^	88.7 (3)	N1—C8—H8A	109.3
N4—Zn1—N10^ii^	88.0 (3)	C7—C8—H8A	109.3
N3^i^—Zn1—N7	86.0 (3)	N1—C8—H8B	109.3
N4—Zn1—N7	90.5 (3)	C7—C8—H8B	109.3
N10^ii^—Zn1—N7	91.2 (3)	H8A—C8—H8B	107.9
N3^i^—Zn1—Cl1	93.0 (2)	N3—C9—N1	109.5 (9)
N4—Zn1—Cl1	90.9 (2)	N3—C9—H9	125.3
N10^ii^—Zn1—Cl1	168.89 (19)	N1—C9—H9	125.3
N7—Zn1—Cl1	99.8 (2)	N2—C10—N3	115.1 (9)
N3^i^—Zn1—Cl1^iii^	89.1 (2)	N2—C10—H10	122.5
N4—Zn1—Cl1^iii^	94.2 (2)	N3—C10—H10	122.5
N10^ii^—Zn1—Cl1^iii^	86.6 (2)	N8—C11—N7	114.2 (10)
N7—Zn1—Cl1^iii^	174.7 (2)	N8—C11—H11	122.9
Cl1—Zn1—Cl1^iii^	82.44 (8)	N7—C11—H11	122.9
Zn1—Cl1—Zn1^iii^	97.56 (8)	N9—C12—N7	108.9 (9)
C9—N1—N2	111.4 (9)	N9—C12—H12	125.6
C9—N1—C8	126.6 (9)	N7—C12—H12	125.6
N2—N1—C8	122.0 (8)	N9—C13—C7	110.6 (7)
C10—N2—N1	101.5 (8)	N9—C13—H13A	109.5
C9—N3—C10	102.6 (8)	C7—C13—H13A	109.5
C9—N3—Zn1^iv^	127.0 (7)	N9—C13—H13B	109.5
C10—N3—Zn1^iv^	127.3 (7)	C7—C13—H13B	109.5
C24—N4—C25	103.4 (8)	H13A—C13—H13B	108.1
C24—N4—Zn1	125.4 (7)	O2—C14—C21	109.9 (8)
C25—N4—Zn1	131.1 (7)	O2—C14—C15	106.4 (7)
C24—N5—N6	110.1 (7)	C21—C14—C15	113.6 (7)
C24—N5—C26	127.4 (8)	O2—C14—C26	107.9 (7)
N6—N5—C26	122.4 (8)	C21—C14—C26	107.4 (7)
C25—N6—N5	101.7 (7)	C15—C14—C26	111.5 (8)
C12—N7—C11	102.9 (8)	C16—C15—C20	116.5 (9)
C12—N7—Zn1	126.3 (6)	C16—C15—C14	123.5 (9)
C11—N7—Zn1	128.3 (7)	C20—C15—C14	119.9 (9)
C11—N8—N9	102.4 (8)	C15—C16—F3	120.6 (9)
C12—N9—N8	111.5 (8)	C15—C16—C17	124.4 (10)
C12—N9—C13	126.0 (9)	F3—C16—C17	115.0 (10)
N8—N9—C13	122.1 (7)	C18—C17—C16	115.8 (10)
C22—N10—C23	101.2 (8)	C18—C17—H17	122.1
C22—N10—Zn1^ii^	128.2 (7)	C16—C17—H17	122.1
C23—N10—Zn1^ii^	129.6 (6)	C19—C18—C17	123.1 (9)
C23—N11—N12	103.6 (7)	C19—C18—F4	118.8 (10)
C22—N12—N11	110.1 (7)	C17—C18—F4	118.0 (10)
C22—N12—C21	129.4 (8)	C18—C19—C20	118.6 (10)
N11—N12—C21	120.5 (8)	C18—C19—H19	120.7
C7—O1—H1	109.5	C20—C19—H19	120.7
C14—O2—H2	109.5	C19—C20—C15	121.3 (10)
H3A—O3—H3B	108.7	C19—C20—H20	119.4
H4A—O4—H4B	119.4	C15—C20—H20	119.4
C2—C1—C6	123.2 (12)	N12—C21—C14	112.1 (8)
C2—C1—F1	118.7 (10)	N12—C21—H21A	109.2
C6—C1—F1	118.0 (11)	C14—C21—H21A	109.2
C1—C2—C3	117.2 (10)	N12—C21—H21B	109.2
C1—C2—H2A	121.4	C14—C21—H21B	109.2
C3—C2—H2A	121.4	H21A—C21—H21B	107.9
F2—C3—C2	118.5 (9)	N12—C22—N10	109.7 (8)
F2—C3—C4	117.7 (10)	N12—C22—H22	125.1
C2—C3—C4	123.8 (9)	N10—C22—H22	125.1
C5—C4—C3	114.2 (10)	N11—C23—N10	115.4 (8)
C5—C4—C7	121.1 (8)	N11—C23—H23	122.3
C3—C4—C7	124.7 (8)	N10—C23—H23	122.3
C6—C5—C4	122.7 (9)	N4—C24—N5	110.2 (9)
C6—C5—H5	118.6	N4—C24—H24	124.9
C4—C5—H5	118.6	N5—C24—H24	124.9
C1—C6—C5	118.6 (10)	N6—C25—N4	114.7 (8)
C1—C6—H6	120.7	N6—C25—H25	122.6
C5—C6—H6	120.7	N4—C25—H25	122.6
O1—C7—C4	112.2 (7)	N5—C26—C14	110.9 (7)
O1—C7—C8	109.6 (8)	N5—C26—H26A	109.5
C4—C7—C8	110.1 (8)	C14—C26—H26A	109.5
O1—C7—C13	104.5 (7)	N5—C26—H26B	109.5
C4—C7—C13	111.2 (8)	C14—C26—H26B	109.5
C8—C7—C13	109.0 (7)	H26A—C26—H26B	108.1

Symmetry codes: (i) *x*+1, *y*, *z*; (ii) −*x*+2, −*y*, −*z*+1; (iii) −*x*+3, −*y*+1, −*z*+1; (iv) *x*−1, *y*, *z*.

### Hydrogen-bond geometry (Å, º)

**Table:**

*D*—H···*A*	*D*—H	H···*A*	*D*···*A*	*D*—H···*A*
O1—H1···Cl2^v^	0.82	2.29	3.103 (7)	172
O2—H2···O4^v^	0.82	1.87	2.653 (9)	160
O3—H3*A*···Cl2^vi^	0.85	2.32	3.163 (11)	170
O3—H3*B*···Cl1^iv^	0.85	2.38	3.221 (10)	170
O4—H4*A*···O2^vii^	0.85	2.24	2.784 (9)	122
O4—H4*B*···Cl2	0.85	2.29	3.101 (8)	160

Symmetry codes: (iv) *x*−1, *y*, *z*; (v) −*x*+1, −*y*+1, −*z*+1; (vi) −*x*+1, −*y*+2, −*z*+1; (vii) *x*−1, *y*+1, *z*.
